# Supplementary figures and images for: Propranolol Modulates Cerebellar Circuit Activity and Reduces Tremor
Source: Cells. 2022 Dec 1;11(23):3889. doi: 10.3390/cells11233889 (PMC9740691; doi:10.3390/cells11233889)

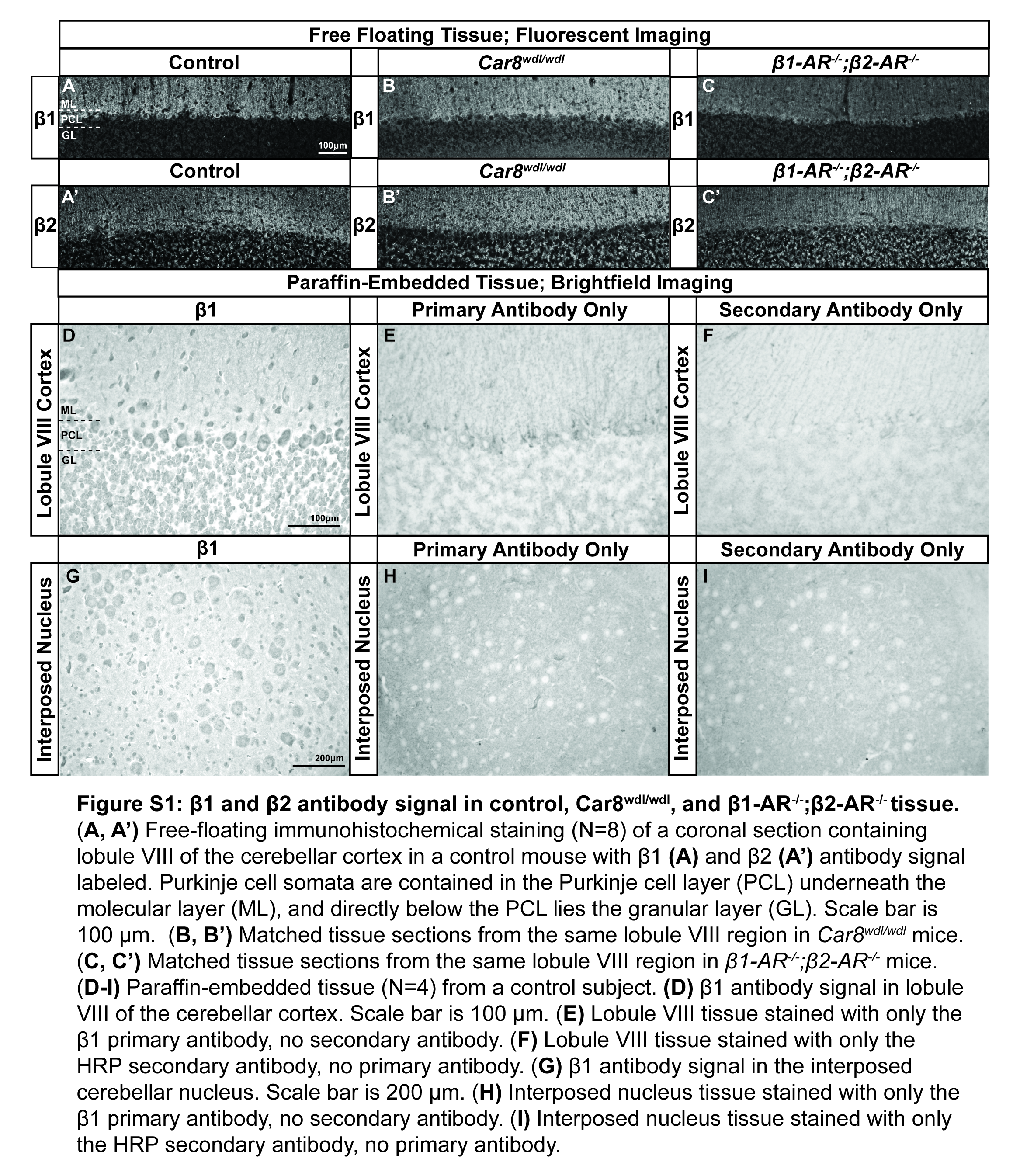

Supplement: Supplementary file 1 [file cells-11-03889-s001.zip › Figure S1.tif]

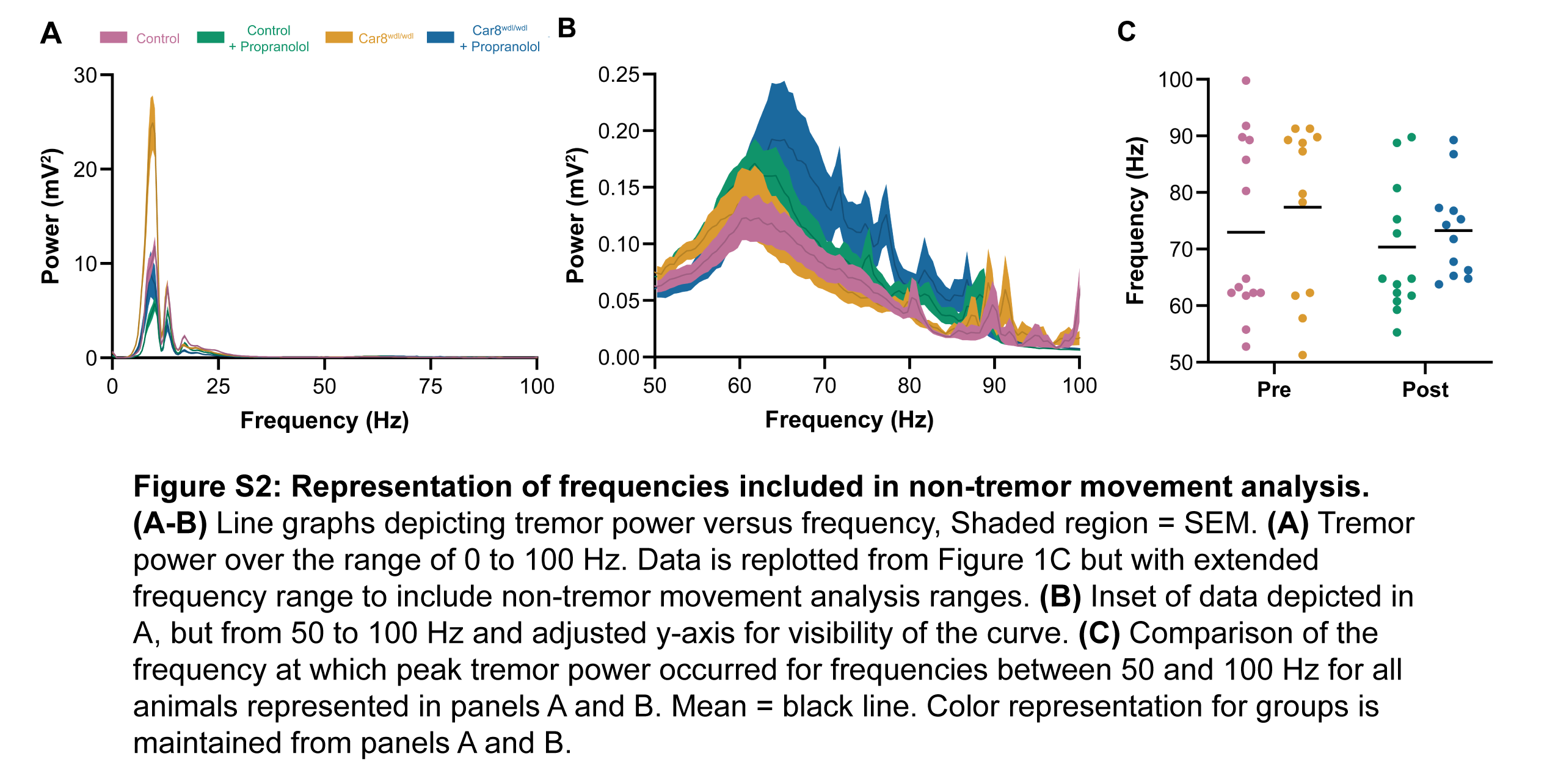

Supplement: Supplementary file 1 [file cells-11-03889-s001.zip › Figure S2.tif]
